# Expression Analysis and Serodiagnostic Potential of Microneme Proteins 1 and 3 in *Eimeria stiedai*

**DOI:** 10.3390/genes11070725

**Published:** 2020-06-29

**Authors:** Wenrui Wei, Nengxing Shen, Jie Xiao, Yuanyuan Tao, Yuejun Luo, Christiana Angel, Xiaobin Gu, Yue Xie, Ran He, Bo Jing, Xuerong Peng, Guangyou Yang

**Affiliations:** 1 Department of Parasitology, College of Veterinary Medicine, Sichuan Agricultural University, Wenjiang 611130, China; wenruiwei1995@stu.sicau.edu.cn (W.W.); shennengxing@stu.sicau.edu.cn (N.S.); xiaojie@stu.sicau.edu.cn (J.X.); taoyuanyuan@stu.sicau.edu.cn (Y.T.); zhengyl2020@stu.sicau.edu.cn (Y.L.); zhengry2019@stu.sicau.edu.cn (C.A.); 13720@sicau.edu.cn (X.G.); 14265@sicau.edu.cn (Y.X.); ranhe1991@sicau.edu.cn (R.H.); zhangcy2018@stu.sicau.edu.cn (B.J.); 2 Department of Veterinary Parasitology, Faculty of Veterinary Sciences, Shaheed Benazir Bhutto University of Veterinary and Animal Sciences, Sakrand 67210, Sindh, Pakistan; 3 Department of Chemistry, College of Life and Basic Science, Sichuan Agricultural University, Wenjiang 611130, China; 10533@sicau.edu.cn

**Keywords:** *Eimeria stiedai*, rabbit, hepatic coccidiosis, microneme proteins, quantitative real-time PCR, indirect ELISA

## Abstract

*Eimeria stiedai* is an apicomplexan protozoan parasite that invades the liver and bile duct epithelial cells in rabbits and causes severe hepatic coccidiosis, resulting in significant economic losses in the domestic rabbit industry. Hepatic coccidiosis lacks the typical clinical symptoms and there is a lack of effective premortem tools to timely diagnose this disease. Therefore, in the present study we cloned and expressed the two microneme proteins i.e., microneme protein 1 (*EsMIC1*) and microneme protein 3 (*EsMIC3*) from *E. stiedai* and used them as recombinant antigens to develop a serodiagnostic method for an effective diagnosis of hepatic coccidiosis. The cDNAs encoding *EsMIC1* and *EsMIC3* were cloned and the mRNA expression levels of these two genes at different developmental stages of *E. stiedai* were determined by quantitative real-time PCR analysis (qRT-PCR). The immunoreactivity of recombinant EsMIC1 (rEsMIC1) and EsMIC3 (rEsMIC3) proteins were detected by Western blotting, and indirect enzyme-linked immunosorbent assays (ELISAs) based on these two recombinant antigens were established to evaluate their serodiagnostic potential. Our results showed that the proteins encoded by the ORFs of *EsMIC1* (711 bp) and *EsMIC3* (891 bp) were approximately 25.89 and 32.39 kDa in predicted molecular weight, respectively. Both *EsMIC1* and *EsMIC3* showed the highest mRNA expression levels in the merozoites stage of *E. stiedai*. Western blotting analysis revealed that both recombinant proteins were recognized by *E. stiedai* positive sera, and the indirect ELISAs using rEsMIC1 and rEsMIC3 were developed based on their good immunoreactivity, with 100% (48/48) sensitivity and 97.9% (47/48) specificity for rEsMIC1 with 100% (48/48) sensitivity and 100% (48/48) specificity for rEsMIC3, respectively. Moreover, rEsMIC1- and rEsMIC3-based indirect ELISA were able to detect corresponding antibodies in sera at days 6, 8, and 10 post *E. stiedai* infection, with the highest positive diagnostic rate (62.5% (30/48) for rEsMIC1 and 66.7% (32/48) for rEsMIC3) observed at day 10 post infection. Therefore, both *EsMIC1* and *EsMIC3* can be used as potential serodiagnostic candidate antigens for hepatic coccidiosis caused by *E. stiedai*.

## 1. Introduction

Rabbit coccidiosis is a severe protozoal disease caused by infection of genus *Eimeria* in the intestines and liver of rabbits, it is one of the most common and highly contagious diseases in rabbits which leads to severe economic losses in domestic rabbit industry [1,2,3]. *Eimeria stiedai* is considered to be one of the most pathogenic *Eimeria* species infecting rabbits, which predominantly invades the epithelial cells of the liver and bile duct, causing severe hepatic coccidiosis characterized by cirrhosis and intensive cholestasis [4,5,6]. While the sporulated oocysts in the environment are ingested by rabbits, the oocyst walls rupture under the stimulation of gastric juice, bile and pancreatic juice. Then sporozoites escape and invade the epithelial cells of the liver and bile duct where merogony occurs. During merogony the merozoites continue to increase until they complete four generations, then gradually form the gametocytes. At the gametogony stage, the gametocytes combine with each other to form zygotes and eventually develop into oocysts. The oocysts will sporulate under suitable conditions and continue to infect rabbits [7]. Affected rabbits mainly show clinical symptoms such as diarrhea, dehydration, growth retardation, and even death in conditions of exacerbating infections [7,8]. Due to the high morbidity and mortality rates caused by hepatic coccidiosis, *E. stiedai* is regarded as one of the most harmful pathogens in rabbitries which severely affects the development of the domestic rabbit industry [9,10]. 

Given the fact that hepatic coccidiosis mostly tends to be of chronic nature and the affected rabbits lack obvious clinical symptoms [11], an accurate diagnosis of this disease during growing and breeding stages is often very difficult, resulting in aggravating the condition of affected rabbits. In view of the strong pathogenicity of *E. stiedai* and its severe impact on the domestic rabbit industry, use of a reliable diagnostic method to diagnose affected rabbits is the basis for effective prevention and control of hepatic coccidiosis [12,13]. However, an accurate diagnosis of hepatic coccidiosis is generally based on the postmortem examination of the affected organs i.e., liver and bile duct, and there is a dearth of studies reporting efficient methods for premortem clinical diagnosis of this disease currently [14]. Therefore, the establishment of a serological diagnostic method for an accurate premortem diagnosis of hepatic coccidiosis is of great significance for the prevention and control of this disease.

The microneme proteins are a group of proteins secreted by the microneme organelles, which are located at the apical tip of the invading stage of apicomplexan protozoa such as *Toxoplasma gondii*, *Plasmodium falciparum*, *Neospora caninum* and *Eimeria tenella* [15,16,17,18]. Infection of apicomplexan protozoa is an extremely rapid and complex process that involves gliding motility, adhesion, recognition, and penetration of the host cells by these protozoa. Since the microneme proteins are secreted at the early stages of these processes, they are reported to play an important role in host invasion [19,20]. To date, more than ten microneme proteins have been characterized, which are functionally conserved among the apicomplexan protozoa [21]. However, there are a lack of reports on the microneme proteins of *E. stiedai* currently. Therefore, in the present study we cloned and molecularly expressed *E. stiedai* microneme protein 1 (*EsMIC1*, GeneBank: MN759306) and microneme protein 3 (*EsMIC3*, GeneBank: MN759307), and analyzed their mRNA expression levels at different developmental stages of *E. stiedai*. Subsequently we established the indirect ELISAs based on two recombinant proteins (rEsMIC1 and rEsMIC3) and evaluated their potential for the diagnosis of hepatic coccidiosis. Our results provide a reasonable reference for prevention and control of hepatic coccidiosis in the domestic rabbit industry.

## 2. Materials and Methods

### 2.1. Parasites and Animals

The *Eimeria stiedai* strain used in this study was isolated from the liver and bile duct of a naturally infected New Zealand White rabbit which was obtained from a farm affected by an outbreak of rabbit coccidiosis. The oocysts obtained were sporulated in 2.5% potassium dichromate solution at 28 °C and stored at 4 °C. The pure strain was maintained by passage through coccidia-free New Zealand White rabbits (four-weeks old) at six-month intervals. Unsporulated oocysts and sporulated oocysts for mRNA expression analyses were purified and stored in liquid nitrogen, merozoites and gametocytes were collected and purified from bile duct of rabbits at corresponding developmental time post infection with sporulated oocysts (*c.* 8 × 10^4^ oocysts per rabbit) [2].

Forty-eight coccidia-free New Zealand White rabbits (four-weeks old, 0.5–0.7 kg body weight) were raised at the Department of Parasitology, College of Veterinary Medicine, Sichuan Agricultural University and kept strictly in a coccidia-free environment according to the method described by Shi et al. [22]. The young rabbits were weaned at the age of 18 days, and fed high-temperature sterilized feeding pellets (in-house prepared at this laboratory) in combination with human infant formula till 30 days of age, during which boiled drinking water and feed were provided *ad libitum*. The anticoccidial drugs i.e., diclazuril and decoquinate were used in cross-rotation in water and rabbit cages were regularly flame-sterilized to prevent the contamination of other *Eimeria* species.

### 2.2. Serum

Serum samples (*n* = 48) were isolated from each coccidia-free rabbit and used as negative controls to determine the assay cut-off value and to test the specificity of the indirect ELISA methods established in this study. Feces of coccidia-free rabbits were collected from three to four weeks of age and examined daily using the saturated saline floating method to ensure that no infection of other *Eimeria* species existed. Positive sera against *E. stiedai* (48 samples) were isolated from rabbits experimentally infected with sporulated oocysts (*c.* 8 × 10^4^ oocysts per rabbit) for a four-week period with obvious liver lesions as determined by necropsy. These serum samples were used to test the sensitivity of the indirect ELISA methods. In addition, serum samples (*n* = 48) isolated from same experimentally infected rabbits at days 6, 8, and 10 post infection (PI) were used to evaluate the early diagnostic potential of recombinant antigens. Positive sera against *Sarcoptes scabiei* (three samples, confirmed by visible skin lesions in hind limbs and mites in skin scrapings) and *Eimeria* spp. (three samples, mixed infection which excluded *E. stiedai* and confirmed by necropsy) were isolated from naturally infected rabbits in two different rabbit farms in the Sichuan Province of China. These serum samples were used for Western blotting to verify cross-reactivity of recombinant antigens with other common parasites in rabbits. All serum samples were stored at −20 °C for subsequent analyses.

### 2.3. Bioinformatics Analyses, Cloning and Sequencing of EsMIC1 and EsMIC3

The open reading frames (ORF) and the deduced amino acid sequences of *EsMIC1* and *EsMIC3* were determined using the ORF Finder [23]. The potential presence of signal peptides and transmembrane regions were predicted using SignalP 5.0 Server [24] and TMHMM Server v. 2.0. [25], respectively. The tools on the ExPASy ProtParam tool [26] were used to predict the molecular weight (MW) and isoelectric point (pI) of *EsMIC1* and *EsMIC3*. 

Total RNA was extracted from sporulated oocysts of *E. stiedai* (*c.* 1 × 10^5^ oocysts, washed repeatedly with physiological saline for five times) using an RNA extraction kit (MiniBEST Universal RNA Extraction Kit, TaKaRa, Japan) and reverse transcribed into cDNA using a reverse transcription system kit (PrimeScriptTM RT reagent Kit with gDNA Eraser, TaKaRa, Japan). The complementary double-stranded cDNA was stored at −80 °C and used as a template to amplify the full coding sequence of *EsMIC1* and *EsMIC3*. Primers for *EsMIC1* and *EsMIC3* were designed using Primer 5.0 software based on our transcriptome data of *E. stiedai* as follows: *EsMIC1*-Forward, 5′-CGGGATCCGCAAGTATGTGTGGGTCGGTT-3′, with a *BamH*I restriction site (underlined); *EsMIC1*-Reverse, 5′-CCAAGCTTTCAGTCAGTGCGGCGGA-3′, with a *Hind*III restriction site (underlined); *EsMIC3*-F, 5′-CGGGATCCTATGAGGGATTGCATCTCGATC-3′, with a *BamH*I restriction site (underlined); *EsMIC3*-R, 5′-CCAAGCTTTCACAAGCCCGCCCCTA-3′, with a *Hind*III restriction site (underlined). *EsMIC1* and *EsMIC3* were amplified by PCR using the cycling conditions as follows: initial denaturation at 94 °C for 5 min; followed by 35 cycles of amplification at 94 °C for 45 s, 56 °C (*EsMIC1*)/59 °C (*EsMIC3*) for 45 s and 72 °C for 1 min; and a final extension step at 72 °C for 10 min. After agarose gel-purification using a QIAquick Gel Extraction Kit (Tiangen, Beijing, China), the PCR products of *EsMIC1* and *EsMIC3* were cloned into the vector pMD19-T (TaKaRa, Dalian, China) and transformed into *Escherichia coli* DH5α (Tiangen, Beijing, China), and subsequently sent for sequencing (Invitrogen, Shanghai, China).

### 2.4. mRNA Expression Analyses of EsMIC1 and EsMIC3 at Different Developmental Stages of E. stiedai

Total RNA from unsporulated oocysts, sporulated oocysts, merozoites, and gametocytes of *E. stiedai* was extracted and the corresponding cDNAs were prepared as described above. The mRNA expression profiles of *EsMIC1* and *EsMIC3* at four different developmental stages of *E. stiedai* were determined using quantitative real-time PCR (qRT-PCR) and 18S ribosomal RNA fragment of *E. stiedai* was used as an internal control for normalization. The 20 μL reactions were performed as per the instructions of SsoAdvanced Universal SYBR Green (Bio-Rad Laboratories, Hercules, CA, USA) with 2 μL of cDNA as a template. The reaction conditions of qRT-PCR were as follows: an initial denaturation at 95 °C for 5 min; followed by 40 cycles of denaturation at 95 °C for 5 s, extension at 60 °C (conformity for both two genes) for 30 s; and a final formation of dissociation curve step at 95 °C for 15 s, 60 °C for 1 min, 95 ° C for 15 s. All primers for qRT-PCR are listed in Table 1. The expression profiles were calculated using the 2^−ΔΔCT^ method and both *EsMIC1* and *EsMIC3* were tested in quadruplicate.

### 2.5. Expression and Purification of rEsMIC1 and rEsMIC3

The correct amplified fragment of *EsMIC1* and *EsMIC3* were digested with *BamH*I and *Hind*III (TaKaRa, Dalian, China) and cloned into the pET32a(+) vector (Invitrogen, Beijing, China). The constructed expression vectors were transformed into *E. coli* BL21 (DE3) (TIANGEN, Beijing, China) and induced to express the recombinant proteins for 12 h at 37 °C by adding 1 mM isopropyl β-d-1-thiogalactopyranoside (IPTG). The rEsMIC1 and rEsMIC3 were purified by chromatography from inclusion bodies (8 M urea) under denaturing conditions using a Ni-NTA His-tag affinity kit (Bio-Rad Laboratories, Hercules, CA, USA) according to the manufacturer’s instructions. The concentrations of purified rEsMIC1 and rEsMIC3 were determined using a BCA protein kit (Beyotime, Jiangsu, China).

### 2.6. Western Blotting Analyses

The purified rEsMIC1 and rEsMIC3 were subjected to 12% sodium dodecyl sulphate-polyacrylamide gel electrophoresis (SDS-PAGE) separation, and then transferred to nitrocellulose membranes with a constant current (30 mA) at room temperature using a trans-blot SD semi-dry transfer cell (Bio-Rad Laboratories, Hercules, CA, USA) for 35 min. The membranes were washed three times in Tris-buffered saline containing Tween-20 (TBST), blocked with 5% (*w*/*v*) skimmed milk solution in Tris-buffered saline (TBS) at room temperature for 2 h, and then incubated with positive serum of *E. stiedai*, *S. scabiei*, *Eimeria* spp., and negative serum (1:200 *v*/*v* dilution in TBS) at 4 °C for 12 h. After washing four times with TBST, the membranes were incubated with horseradish peroxidase (HRP)-conjugated goat anti-rabbit antibody (Boster, Wuhan, China) (1:1000 *v*/*v* dilution in TBS) at room temperature for 2 h and washed again four times with TBST. An Enhanced HRP-DAB Chromogenic Substrate Kit (TIANGEN, Beijing, China) was used to detect the signals according to the manufacturer’s instructions.

### 2.7. Development of Indirect ELISAs

To determine the optimal serum dilutions and recombinant antigen concentrations of indirect ELISAs, the standard checkerboard titration procedures were performed [27]. Briefly, the purified rEsMIC1 and rEsMIC3 proteins were diluted to six different concentrations (1:20, 1:40, 1:80, 1:160, 1:320, 1:640; i.e., 11.5 μg, 5.75 μg, 2.88 μg, 1.44 μg, 0.72 μg, 0.36 μg per well for rEsMIC1 and 18.5 μg, 9.25 μg, 4.63 μg, 2.31 μg, 1.16 μg, 0.58 μg per well for rEsMIC3) in 0.1 M carbonate buffer (pH 9.6) and coated to polystyrene 96-well microtiter plates (Invitrogen, Shanghai, China) with 100 μL per well, and subsequently incubated overnight at 4 °C. After washing three times with phosphate buffered saline containing Tween-20 (PBST), the plates were blocked with 300 μL 5% (*w*/*v*) skimmed milk solution in phosphate buffered saline (PBS) per well, and incubated at 37 °C for 90 min. The plates were washed again (three times) with PBST, and incubated with 100 μL serum samples diluted to six different concentrations (i.e., 1:20, 1:40, 1:80, 1:160, 1:320, 1:640) in PBS for each well at 37 °C for 1 h. Following washing three times with PBST, 100 μL HRP-labeled goat anti-rabbit IgG (Boster, Wuhan, China) diluted to 1:3000 in PBS was added to each well, and incubated at 37 °C for 1 h. The plates were again thoroughly washed four times with PBST and 100 μL of substrate 3,3,5,5-tetramethylbenzidine (TIANGEN, Beijing, China) was added to each well and the plates were incubated in darkness at 37 °C for 15 min. Finally, 100μL of 2 M H_2_SO_4_ were added to each well to terminate the reaction. After measuring the optical density at 450 nm (OD450) by a microplate reader (Thermo Fisher Scientific, Waltham, MA, USA), the optimal working dilutions of the recombinant antigen and serum were selected according to the P/N value (the dilutions which gave the maximum difference OD450 nm value between positive and negative serum samples). Other working conditions of indirect ELISAs including to recombinant antigens coating time, blocking solution and blocking time, serum samples incubating time, HRP-labeled goat anti-rabbit IgG diluting concentration and incubating time and substrate incubating time were optimized and the optimal reaction conditions of both recombinant antigens were as described above. Under the optimal conditions, 48 negative serum samples collected from coccidia-free rabbits were used to determine the cut-off value of the indirect ELISA, which was calculated as arithmetic mean of the OD450 nm values from negative serum samples plus three standard deviations (SD). 

### 2.8. Sensitivity and Specificity of Indirect ELISAs

To further verify the feasibility of the rEsMIC1- and rEsMIC3-based indirect ELISAs, the sensitivity and specificity were evaluated using positive and negative serum samples against *E. stiedai* (48 samples for each) as described above. The percentage sensitivity was calculated as ELISA positive × 100/true *E. stiedai*-positive, while the percentage specificity was calculated as ELISA negative × 100/true *E. stiedai*-negative.

### 2.9. Early Diagnostic Potential Evaluation of Indirect ELISAs

The serum samples collected from 48 experimentally infected rabbits at days 6, 8, and 10 post *E. stiedai* infection were used to evaluate the early diagnostic potentials of rEsMIC1- and rEsMIC3-based indirect ELISAs. The positive diagnostic rates were determined by contrast of the cut-off value, which was calculated as follows: number of serum samples diagnosed by ELISA × 100/total number of serum samples.

### 2.10. Statistical Analyses

All statistical analyses were performed using IBM SPSS statistics 20.0 (SPSS Inc., Chicago, IL, USA) software. One-way ANOVA followed by a post hoc Duncan’s multiple range test was applied to compare the mRNA expression levels between different developmental stages and the OD450 nm values between different serum groups. A *p* value < 0.05 was considered to be statistically significant. All data are presented as the mean ± standard deviation (SD). The GraphPad Prism software (version 5.01) was used for producing graphs.

### 2.11. Ethics Approval and Consent to Participate

The animal study was reviewed and approved by the Animal Care and Use Committee of Sichuan Agricultural University (SYXK2019-187). All animal procedures used in this study were carried out in accordance with the Guide for the Care and Use of Laboratory Animals (National Research Council, Bethesda, MD, USA) and recommendations of the ARRIVE guidelines [28]. All methods were carried out in accordance with relevant guidelines and regulations.

## 3. Results

### 3.1. Cloning, Sequencing and Bioinformatics Analyses of EsMIC1 and EsMIC3

**Figure 1:**
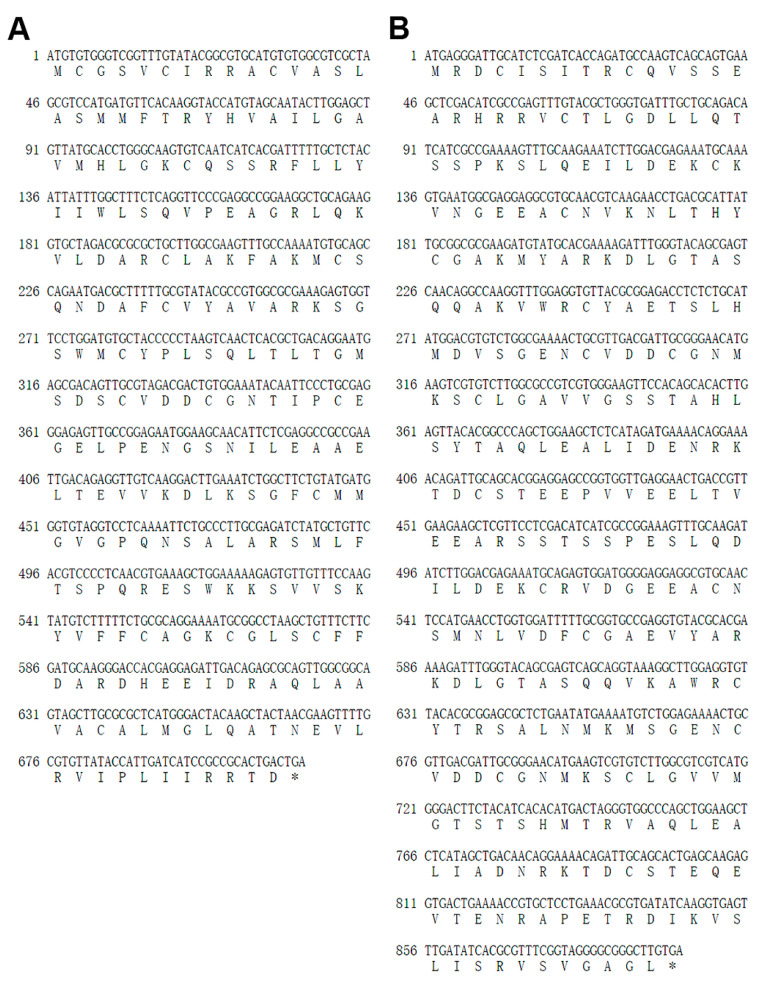
The open reading frame (ORF) and deduced amino acid sequence of *EsMIC1* (GeneBank: MN759306) (**A**) and *EsMIC3* (GeneBank: MN759307) (**B**). Asterisk indicates a stop codon.

The full-length sequences of *EsMIC1* and *EsMIC3* were successfully amplified from the *E. stiedai* cDNA and the correct sequencing results were obtained. The *EsMIC1* cDNA sequence (GeneBank: MN759306) contained a 711 bp open reading frames (ORF) encoding a protein of 236 amino acid residues (Figure 1A) with a predicted molecular weight (MW) of 25.89 kDa and a calculated isoelectric point (pI) of 8.31, while the *EsMIC3* cDNA sequence (GeneBank: MN759307) contained an ORF of 891 bp encoding a protein of 296 amino acid residues (Figure 1B) with a predicted MW of 32.39 kDa and a calculated pI of 5.08. In addition, no predicted signal peptide and transmembrane regions were found in either protein.

### 3.2. mRNA Expression of EsMIC1 and EsMIC3 at Different Developmental Stages of E. stiedai 

**Figure 2:**
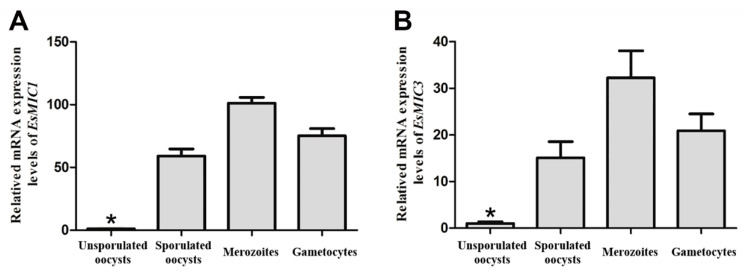
Relative mRNA expression levels of *EsMIC1* (**A**) and *EsMIC3* (**B**) at different developmental stages of *E. stiedai*. Data are presented as mean and standard deviation (SD) of quadruplicate experiments. The expression profiles were calculated using the 2^−ΔΔCT^ method and normalized to the levels of the 18S ribosomal RNA fragment of *E. stiedai*. Asterisks indicate statistically significant differences in the expression levels between unsporulated oocyst stage and other developmental stages (* *p* < 0.05).

The mRNA expression profiles of *EsMIC1* and *EsMIC3* at different developmental stages (unsporulated oocysts, sporulated oocysts, merozoites, and gametocytes) of *E. stiedai* were determined using quantitative real-time PCR (qRT-PCR). The results showed that both *EsMIC1* and *EsMIC3* were transcribed in all four developmental stages of *E. stiedai*, and the mRNA expression levels of both genes were ordered as follows: merozoites > gametocytes > sporulated oocysts > unsporulated oocysts (Figure 2). The relative mRNA expression levels of *EsMIC1* in the merozoites stage were 1.4-fold, 1.7-fold and 103-fold higher compared to the gametocytes, sporulated oocysts, and unsporulated oocysts, respectively. While the relative mRNA expression levels of *EsMIC3* in merozoites stage were 1.6-fold, 2.1-fold, and 32-fold higher compared to the gametocytes, sporulated oocysts, and unsporulated oocysts, respectively. 

### 3.3. Expression and Purification of rEsMIC1 and rEsMIC3

**Figure 3:**
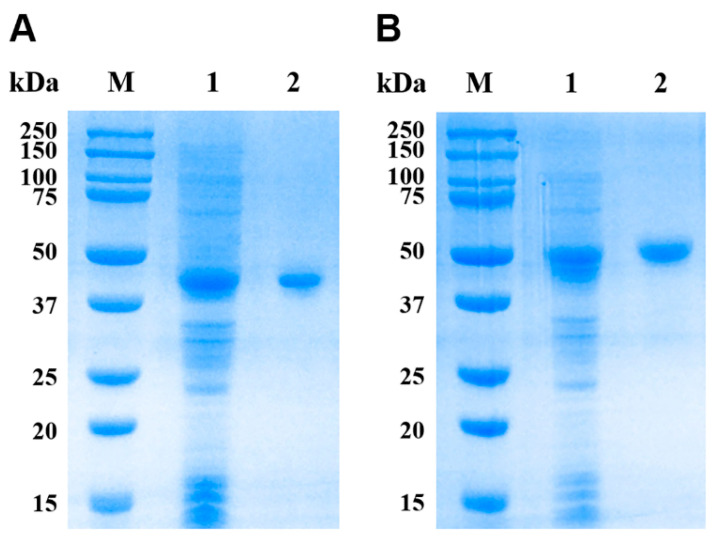
Expression and purification of rEsMIC1 and rEsMIC3. (**A**) Lane M: protein molecular weight markers; Lane 1: IPTG-induced rEsMIC1 in *Escherichia coli* BL21 (DE3); Lane 2: purified rEsMIC1 (5 µg). (**B**) Lane M: protein molecular weight markers; Lane 1: IPTG-induced rEsMIC3 in *Escherichia coli* BL21 (DE3); Lane 2: purified rEsMIC3 (5 µg).

The expression vectors pET32a(+)-*EsMIC1* and pET32a(+)-*EsMIC3* were successfully constructed, and both rEsMIC1 (~45 kDa) and rEsMIC3 (~52 kDa) were successfully expressed in inclusion bodies by *E. coli* BL21 (DE3) (Figure 3, lane 1). Excluding an approximately 20 kDa epitope tag fusion peptide encoded by pET32a(+). The pET-32(+) series is designed for cloning and high-level expression of peptide sequences fused with the 109 aa thioredoxin protein (Trx) Tag, cleavable histidine (His) Tag and S Tag sequences. These tags were necessary for further detection and purification of recombinant proteins, the molecular weights of rEsMIC1 and rEsMIC3 were similar to those predicted from their amino acid sequences, indicating the successful expression of these two recombinant proteins. After purification and concentration by Ni-chelating column chromatography, the single bands of rEsMIC1 and rEsMIC3 were obtained (Figure 3, lane 2). The concentrations of rEsMIC1 and rEsMIC3 were observed to be 2.3 and 3.7 mg/mL, respectively.

### 3.4. Western Blotting Analyses

**Figure 4:**
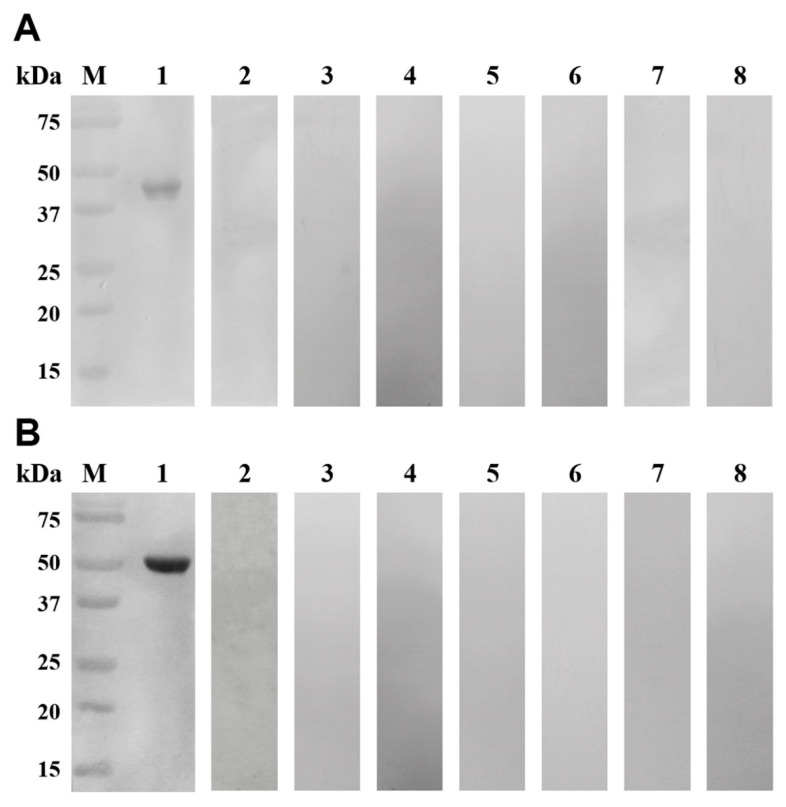
Western blot analyses of rEsMIC1 and rEsMIC3. (**A**) Lane M: protein molecular weight markers; Lane 1: purified rEsMIC1 (5 µg) probed with positive serum sample against *E. stiedai*; Lane 2: purified rEsMIC1 (5 µg) probed with negative control rabbit serum sample; Lanes 3–5: purified rEsMIC1 (5 µg) probed with positive serum samples against *S. scabiei*; Lanes 6–8: purified rEsMIC1 (5 µg) probed with positive serum samples against *Eimeria* spp. (**B**) Lane M: protein molecular weight markers; Lane 1: purified rEsMIC3 (5 µg) probed with positive serum sample against *E. stiedai*; Lane 2: purified rEsMIC3 (5 µg) probed with negative control rabbit serum sample; Lanes 3–5: purified rEsMIC3 (5 µg) probed with positive serum samples against *S. scabiei*; Lanes 6–8: purified rEsMIC3 (5 µg) probed with positive serum samples against *Eimeria* spp.

Western blotting analyses showed that the purified rEsMIC1 and rEsMIC3 were recognized by positive serum against *E. stiedai* and presented a single band entirely (Figure 4, lane 1). No bands were observed in the negative controls (Figure 4, lane 2), indicating the strong reactivity and good immunoreactivity of these two recombinant proteins. Moreover, no bands were observed when positive serum against *S. scabiei* and *Eimeria* spp. were used to recognize the rEsMIC1 and rEsMIC3, indicating the absence of cross-reactivity between these two recombinant proteins and positive serum against *S. scabiei* and *Eimeria* spp. (Figure 4, lanes 3–5 and 6–8). Furthermore, the difference in intensity of bands between rEsMIC1 and rEsMIC3 may be mainly attributed to the difference in reaction intensity between serum samples and proteins (Figure 4, lane 1).

### 3.5. Establishment of Indirect ELISA

In order to establish an indirect ELISA method for rabbit hepatic coccidiosis, we determined the optimal reaction conditions of rEsMIC1 and rEsMIC3 as recombinant antigens using the checkerboard titration method. The optimal antigen concentrations of rEsMIC1 and rEsMIC3 were 1.15 and 0.74 μg per well, respectively, and the optimal serum dilution was 1:160 for both recombinant antigens, which gave the highest P/N value. Under the optimal reaction conditions, a total of 48 negative serum samples were used to determine the cut-off values, which were calculated as 0.309 (rEsMIC1-based ELISA; mean = 0.188, SD = 0.0403) and 0.253 (rEsMIC3-based ELISA; mean = 0.157, SD = 0.0319). Serum samples with OD450 nm value ≥0.309 (rEsMIC1-based ELISA) or 0.253 (rEsMIC3-based ELISA) were therefore considered as *E. stiedai* positive, otherwise they were considered as negative.

### 3.6. Sensitivity and Specificity of Indirect ELISA

**Figure 5:**
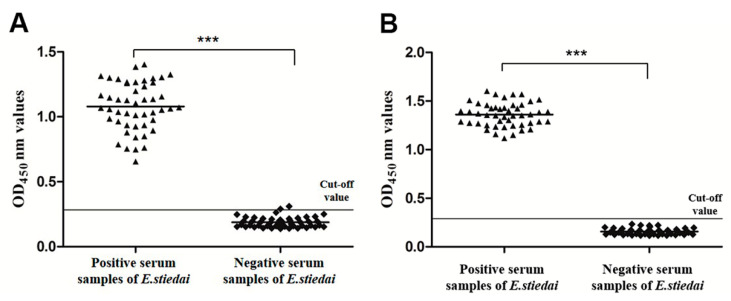
Detection of positive and negative serum samples against *E. stiedai* using established indirect ELISA based on rEsMIC1 and rEsMIC3. (**A**) Sensitivity and specificity of the rEsMIC1-based ELISA. The horizontal line represents the cut off value (Cut-Off = 0.309), statistically significant differences were observed between positive serum samples and negative control (*** *p* < 0.01); (**B**) Sensitivity and specificity of the rEsMIC3-based ELISA. The horizontal line represents the cut off value (Cut-Off = 0.253), statistically significant differences were observed between positive serum samples and negative control (*** *p* < 0.01).

To determine the sensitivity and specificity of rEsMIC1- and rEsMIC3-based indirect ELISAs, positive and negative serum samples (48 for each) against *E. stiedai* were tested under the optimal conditions. The results showed that for the rEsMIC1-based indirect ELISA, all 48 positive serum samples were correctly detected and one out of 48 negative serum samples was detected as positive based on the cut-off value (0.309), and hence the sensitivity and specificity of the rEsMIC1-based ELISA were calculated as 100% (48/48) and 97.9% (47/48), respectively (Figure 5A). Meanwhile, for the rEsMIC3-based indirect ELISA, all serum samples (positive and negative) were correctly detected based on the cut-off value (0.253), indicating that the sensitivity and specificity of the rEsMIC3-based ELISA were both calculated as 100% (48/48 for each positive and negative samples) (Figure 5B). We also observed the significant differences in mean OD values between positive and negative serum samples for both rEsMIC1-based (*F*_(1,94)_ = 1050.080, *p* < 0.0001) and rEsMIC3-based (*F*_(1,94)_ = 4450.558, *p* < 0.0001) ELISAs.

### 3.7. Early Diagnostic Potential of Indirect ELISAs 

**Figure 6:**
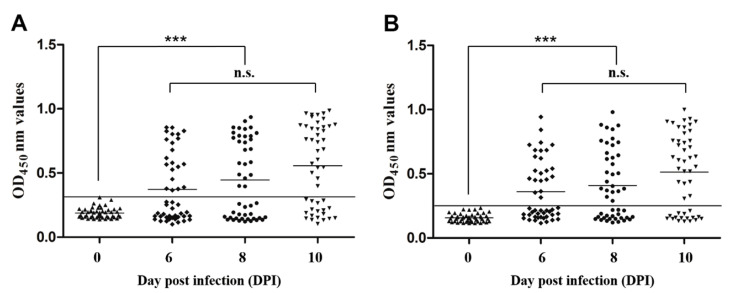
Detection of serum samples on days 0, 6, 8, and 10 post *E. stiedai* infection using established indirect ELISAs based on rEsMIC1 and rEsMIC3. (**A**) Early diagnosis testing of rEsMIC1-based ELISAs. The horizontal line represents the critical value (Cut-Off = 0.309). Forty-eight serum samples collected from experimentally infected rabbits at days 6, 8, and 10 post infection (PI) were detected and statistically significant differences were observed between negative control (day 0 PI) and days 6,8, and 10 PI groups (*** *p* < 0.01). (**B**) Early diagnosis testing of the rEsMIC3-based ELISA. The horizontal line represents the critical value (Cut-Off = 0.253). Forty-eight serum samples collected from experimentally infected rabbits at days 6, 8, and 10 PI groups were detected and statistically significant differences were observed between negative control (day 0 PI) and days 6, 8, and 10 PI groups (*** *p* < 0.01).

To evaluate the early diagnostic potential of the rEsMIC1- and rEsMIC3-based indirect ELISAs, the serum samples collected from 48 experimentally infected rabbits at days 6, 8, and 10 post *E. stiedai* infection were used. The results showed that for the indirect ELISA based on rEsMIC1, the positive diagnosis rates at days 6, 8, and 10 post infection (PI) were 43.8% (21/48), 52.1% (25/48), and 62.5% (30/48), respectively (Figure 6A). Furthermore, for the indirect ELISA method based on rEsMIC3, the positive diagnosis rates at days 6, 8, and 10 PI were 45.8% (22/48), 56.3% (27/48), and 66.7% (32/48), respectively (Figure 6B). In addition, significant differences in the mean OD values were observed between the negative control (day 0 PI) and days 6, 8, and 10 PI groups for both rEsMIC1-based (*F*_(3,188)_ = 17.935, *p* < 0.0001) and rEsMIC3-based (*F*_(3,188)_ = 20.094, *p* < 0.0001 ) ELISAs.

## 4. Discussion

Microneme proteins are a unique group of secreted proteins peculiar to the parasites belonging to the phylum Apicomplexa including *Eimeria*, which are mainly involved in the process of parasite adhesion and invasion of the host cells [29,30]. In the early stages of parasite interaction with the host cells, microneme proteins are first secreted from the apex of the merozoite and promote the adhesion by recognizing the receptors on the host cell membrane, thus making them crucial contributors to the invasion process of parasites [31]. Due to these features, previous studies have mainly focused on microneme proteins as recombinant diagnostic antigens, vaccine candidate antigens, and anti-parasitic drug targets in the control of several apicomplexan parasite infections, including *Toxoplasma gondii* [32,33,34], *Plasmodium falciparum* [35,36], *Neospora caninum* [37,38], and *Eimeria tenella* [39,40,41].

Microneme protein 1 (MIC1), the earliest reported microneme protein in *Eimeria tenella*, is a glycoprotein G-related protein containing a TSP region consisting of five repeating hydrophilic amino acids, a conserved transmembrane region (CTD), and a long terminal hydrophobic amino acid region (CTR) [42,43]. Microneme protein 3 (MIC3) was first identified in *E. tenella* by Labbe et al. [44], which contains seven tandem repeat regions i.e., four highly conserved intramembrane repeat regions and three more mutated outer repeat regions. However, there are no related reports on microneme proteins of *Eimeria* spp. infecting rabbits, in particular *E. stiedai*. Therefore, in the present study, we focused on two microneme proteins (MIC1 and MIC3), which have been identified in *E. tenella*. The sequences of the two *E. stiedai* genes i.e., *EsMIC1* and *EsMIC3* were obtained from our transcriptome data, and rEsMIC1 and rEsMIC3 were successfully cloned and expressed.

In the present study, the mRNA expression profiles of *EsMIC1* and *EsMIC3* at different developmental stages of *E. stiedai* were evaluated by qRT-PCR. Both *EsMIC1* and *EsMIC3* showed the highest expression levels in the merozoites, suggesting that these two microneme proteins might play important roles in the merogony stages of *E. stiedai*. The results of mRNA expression analyses in our study are consistent with the findings of Novaes et al. [45], who reported that microneme proteins showed a high expression level during the merozoite stages of *Eimeria* spp. in fowl. Meanwhile, both MIC1 and MIC3 proteins of *E. tenella* have been implicated in the process of invasion of the host cells and the merogony of parasites [44,46,47]. Therefore, both *EsMIC1* and *EsMIC3* may be the potential diagnostic and vaccine candidate antigens against hepatic coccidiosis.

Rabbit coccidiosis is currently a prevalent disease worldwide [48,49,50], and has caused serious economic losses to the rabbit industry in many countries. Hepatic coccidiosis has the highest pathogenicity and the most serious epidemic and can cause severe damage to the liver and even death in young rabbits during extreme infections, however, the adults can become carriers and serve as a source of infection [14]. At present, an accurate diagnosis of hepatic coccidiosis requires a comprehensive postmortem examination of the infected rabbits, and there is still no effective premortem diagnostic method available. One previous study used the *E. stiedai* oocysts as the natural diagnostic antigens for diagnosis [51]. However, the utilization of oocyst as a diagnostic antigen has a series of inherent problems, for instance, difficulty in collecting and preparing the standard antigens, determination of sources and dosages of antigens, and potential obstruction in the promotion and application of antigens [52]. On the contrary compared to the natural oocyst antigens, recombinant antigens have the advantage of high reproducibility, strong specificity, source stability and convenient mass production [53]. However, studies focusing on the potential use of recombinant antigens as the serological diagnostic tools against rabbit coccidiosis are lacking [54]. Holec et al. [32] established an indirect ELISA method using the recombinant MIC1 protein of *T. gondii* as a diagnostic antigen to test the serum of patients infected with *T. gondii*, the results showed that the sensitivity and specificity of the method were 94.4% (68/72) and 88.9% (21/24), respectively, indicating a good diagnostic value of rTgMIC1. Similarly, in another study [55], the recombinant protein MIC3 of *T. gondii* was used as a diagnostic antigen to establish a latex agglutination test (LAT), and to evaluate the efficacy of the diagnosis of *T. gondii* antibodies in swine sera. It was shown that rTgMIC3 had a strong agglutination reaction with *T. gondii* positive serum, and no cross-reaction was observed between rTgMIC3 and antibodies of other common pathogens in swine. It is worth mentioning that evidence from both of the above studies [32,55] reasonably indicates that MIC1 and MIC3 have a good diagnostic value for the apicomplexan infections, and can be used as recombinant antigens for diagnosis. In the present study, rEsMIC1 and rEsMIC3 were used as recombinant diagnostic antigens to establish indirect ELISA methods for serological diagnosis of hepatic coccidiosis. Our results showed that the indirect ELISA methods based on these two recombinant antigens had 100% (48/48) sensitivity and 97.9% (47/48) specificity for rEsMIC1, while 100% sensitivity (48/48) and specificity (48/48) for rEsMIC3. These findings indicate that both rEsMIC1 and rEsMIC3 have a good diagnostic value and can be used as the candidate diagnostic antigens for clinical diagnosis of hepatic coccidiosis. However, in view of that rabbits can be infected by other intestinal *Eimeria* spp. and our study only investigated the cross-reactivity with these *Eimeria* spp. by Western blotting, thus potential cross-reactivity with intestinal *Eimeria* spp. of rabbits should be further studied in more detail prior to a broader use of rEsMIC1 and rEsMIC3 as diagnostic antigens. In addition, our study only established indirect ELISAs based on rEsMIC1 and rEsMIC3 and found out the serodiagnostic potential of these two antigens, the methods should be further used to test a larger number of field sera for estimate diagnostic sensitivity and specificity under field conditions and investigate the prevalence of *E. stiedai*.

## 5. Conclusions

In the present study, microneme protein 1 and 3 of *E. stiedai* were cloned, expressed and characterized for the first time. qRT-PCR analysis revealed the highest relative mRNA expression levels of *EsMIC1* and *EsMIC3* at the merozoite stage of *E. stiedai*. At the same time, the indirect ELISA methods based on rEsMIC1 and rEsMIC3 were established and confirmed to have good sensitivity and specificity, indicating that both recombinant proteins can be used as suitable potential candidate diagnostic antigens for clinical diagnosis of hepatic coccidiosis. The results of our study provide a reference for the establishment of a serological diagnosis method for rabbit coccidiosis, and also lay a reasonable foundation for the prevention and control of hepatic coccidiosis in the domestic rabbit industry.

## Figures and Tables

**Table 1:** Primers used for quantitative real-time PCR (qRT-PCR) amplification of *EsMIC1* and *EsMIC3*.

Gene	qRT-PCR Primer Sequences	Size (bp)
*EsMIC1*	F: 5′-TGACAGGAATGAGCGACAGTTGC-3′	99
	R: 5′-CGGCGGCCTCGAGAATGTTG-3′	
*EsMIC3*	F: 5′-CTCGATCACCAGATGCCAAGTCAG-3′	141
	R: 5′-CACGCCTCCTCGCCATTCAC-3′	
*18S ribosomal RNA*	F: 5′-GGAGTTGACGAAAGGGCACCAC-3′	113
	R: 5′-GCCATGCACCACCACCCATAG-3′	
